# Cytokines and Leukocytes Subpopulations Profile in SARS-CoV-2 Patients Depending on the CT Score Severity

**DOI:** 10.3390/v13050880

**Published:** 2021-05-11

**Authors:** Elżbieta Rutkowska, Iwona Kwiecień, Magdalena Żabicka, Artur Maliborski, Agata Raniszewska, Krzysztof Kłos, Weronika Urbańska, Izabella Klajnowicz, Piotr Rzepecki, Andrzej Chciałowski

**Affiliations:** 1 Laboratory of Hematology and Flow Cytometry, Department of Internal Medicine and Hematology, Military Institute of Medicine, 04-141 Warsaw, Poland; ikwiecien@wim.mil.pl (I.K.); araniszewska@wim.mil.pl (A.R.); 2 Department of Radiology, Military Institute of Medicine, 04-141 Warsaw, Poland; mzabicka@wim.mil.pl (M.Ż.); amaliborski@wim.mil.pl (A.M.); 3 Department of Infectious Diseases and Allergology, Military Institute of Medicine, 04-141 Warsaw, Poland; kklos@wim.mil.pl (K.K.); wurbanska@wim.mil.pl (W.U.); iklajnowicz@wim.mil.pl (I.K.); achcialowski@wim.mil.pl (A.C.); 4 Department of Internal Medicine and Hematology, Military Institute of Medicine, 04-141 Warsaw, Poland; przepecki@wim.mil.pl

**Keywords:** SARS-CoV-2, leukocyte, lymphocyte, T regulatory cell, cytokines, IL-6, TNF-α, IL-1β, computed tomography, CT severity score

## Abstract

The role of the adaptive microenvironment components in severe acute respiratory syndrome coronavirus 2 (SARS-Cov-2) infection is widely researched, but remains unclear. Studying the common dynamics of adaptive immune response changes can help understand the pathogenesis of coronavirus disease 2019 (COVID-19), especially in critical patients. The aim of the present study was to determine the cytokines concentration and leukocyte subpopulations profiles in the severe COVID-19 (*n* = 23) and critical (*n* = 18) COVID-19 group distinguished by the computed tomography (CT) severity score. We observed lower percentage of lymphocyte subpopulation, higher neutrophils to lymphocytes ratio (NLR) and higher IL-6 concentration in critical COVID-19 group than in severe group. CT severity score was negative correlated with proportion of lymphocytes, lymphocytes T, CD4+ cells, Treg cells and NK cells and positive correlated with neutrophils, NLR, and IL-6. In critical group more correlations between cytokines and lymphocytes were observed, mainly between TNF-α, IL-1β and lymphocyte subpopulations. The collective assessment of the cytokine profile, leukocyte subpopulations and the CT severity score can help to characterize and differentiate patient in advanced COVID-19 than the study of single parameters. We have shown that the interconnection of elements of the adaptive microenvironment can play an important role in critical COVID-19 cases.

## 1. Introduction

Coronavirus disease 2019 (COVID-19) is presented as a newly diagnosed pneumonia and can rapidly develop into acute respiratory syndrome that has led to a global pandemic [1]. Not only the clinical symptoms such as fever, cough, dyspnea are characteristic features of COVID-19 disease but also some morphological parameters may indicate this infection like lymphopenia, neutrophilia, elevated neutrophil to lymphocyte ratio (NLR), D-dimer concentrations, level of inflammation marker C Reactive Protein (CRP), lactate dehydrogenase (LDH), or elevated reactive lymphocyte (RE-LYMP) parameter together with the cytokine release syndrome, also so-called cytokine storm [2,3,4,5,6] The course of the disease can vary greatly, from asymptomatic to very critical, and may even lead to death [7]. Assessment of severity of the disease is important for fast and effective diagnosis, to be able to implement appropriate procedures and treatment. The standard diagnostic method for suspected SARS-Cov-2 virus infection is the Polymerase Chain Reaction (PCR) test, which detects viral RNA in the sample [8].

Other methods are being sought that may indicate infection with this virus and additionally assess the severity of the disease. Lung lesion severity analysis was developed using computed tomography (CT) to differentiate the form of SARS-CoV-2 disease. It was confirmed that the CT result can be used to quickly and objectively assess the severity of lung involvement in patients with COVID-19 [9,10,11]. A CT scan is not intended to diagnose COVID-19, but it can help determine individual patient management and assess the severity of the disease, complications, or seek an alternative diagnosis. 

A better understanding of clinical features and the identification of reliable laboratory markers of inflammation that can distinguish between mild to moderate and severe to critical infections are needed. The data may also help to better understand the pathogenesis of this infection. However, the exact role that cytokines, leukocytes subsets, and infection-related factors play in disease severity and progression have not yet been established. We examined changes in peripheral blood leukocyte subsets and parallel changes in cytokines level in patients with different disease severity assessed on the basis of the CT severity score to explain the pathogenesis of SARS-CoV-2. 

The aim of our study was to determine the cytokines and leukocyte subpopulations profiles using the CT severity score and to identify the differences between the severe and critical COVID-19 groups.

## 2. Materials and Methods

### 2.1. Patients

A total of 38 patients with SARS-CoV-2 were defined as positive from RT-PCR assay from nasopharyngeal swab specimens according to the WHO guidelines. Patients SARS-CoV-2+ were recruited from 2 November 2020 to 29 January 2021 at the Department of Infectious Diseases and Allergology of Military Institute of Medicine.

There were 16 women and 22 men; mean age: 56.6 ± 13.5 years. Patients were divided into two groups on the basis of lung changes in the course of SARS-CoV-2 infection assessed by CT examination. The first group with advanced lesions consisted of 23 patients – severe COVID-19 group. Second group were 18 patients with critical changes in the lung – critical COVID-19 group. The baseline clinical condition on admission was classified as symptomatic unstable with SpO2 at 91% to 95%, symptomatic unstable with SpO2 ≤90% or acute respiratory distress syndrome. The decision on the treatment regimen was made entirely by the attending physician, taking into account the current knowledge and recommendations of the Polish Society of Epidemiologists and Infectiologist [12]. From the analyzed patients, two patients were treated in the ICU. One patient died. There was no co-infection in the analyzed group of patients. The mean hospitalization was 20.5 +/− 14 days.

Clinical characteristics of all COVID-19 patients were presented in Table 1.

### 2.2. CT Severity Score

The method to identify significant radiological differences between severe and milder cases was based on a scoring system [10,11]. Lung lesion advancement grading in CT was based on visual assessment of the degree of pulmonary tissue involvement by lesions in individual lobes (three lobes of the right lung and two lobes of the left lung) mainly the presence of inflammatory abnormalities, including the presence of ground-glass opacities, crazy paving pattern and pulmonary consolidation. The extent of changes in a whole lobe was evaluated, not individual sections. Two radiologists (M.Ż. and A.M. with 25 and 15 years of experience in lung imaging, respectively) evaluated the extent of changes in lungs and the differences were sorted out by consensus. Percentage changes are presented for each lobe separately, and then all points are summed up. Each lobe could be awarded a CT score from 0 to 5, depending on the percentage of the involved lobe: score 0–0% involvement; score 1—less than 5% involvement; score 2–5% to 25% involvement; score 3–26% to 49% involvement; score 4–50% to 75% involvement; score 5—greater than 75% involvement. The sum of the scores for all lobes gives the score for the severity of lung lesions:

- 0 points—normal lung
- 1–5 points—mild changes
- 6–10 points—moderate changes
- 11–15 points—severe changes
- 16–25 points—critical changes

**Figure A1:**
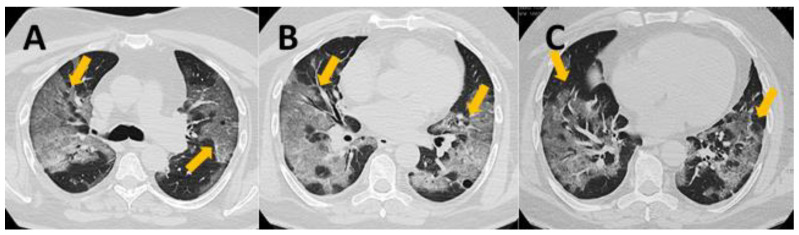
The example of CT changes in patient with critical COVID severity score 20—4 points (50 to 75% of lung tissue involvement) for the each of lung lobe. (**A**) axial scan at the level of upper lobes; (**B**) axial scan at the level of middle lobe on the right side, lingua on the left side and the upper segments of lower lobes; (**C**) axial scan at the level of the basal segments of the lower lobes. The yellow arrows points ground-glass and crazy paving opacifications.

In our study all patients had score more than 11 and were divided into two groups: severe COVID-19 group (*n* = 23) with CT severity score 11–15 and critical COVID-19 group (*n* = 15) with CT severity score 16–25. The example of CT changes in critical COVID patient with severity score 20 was presented on Appendix A Figure A1.

### 2.3. Peripheral Blood Samples

The routine test of white blood cells count (WBC) was performed using a hematological analyzer Sysmex XN-1500 (Sysmex Corp., Kobe, Japan). Peripheral blood (PB) samples were collected from all COVID-19 patients in the time of 24 h after hospital admission and before administration of any antiviral and/or immunosuppressive drug.

### 2.4. Cytokine Measurement

The Luminex^®^ technique was used to detect the cytokine concentration [pg/mL] in the serum samples including: IL-1β, IL-4, IL-5, IL-6, IL-8, IL-10, TNFα according to the manufacturer’s instruction. The Human Magnetic Luminex Assay (R&D Systems^®^, Inc., Minneapolis, MN, USA) on the Luminex platform (Shanghai Universal Biotech Co., Ltd., Shanghai, China) was used.

### 2.5. Flow Cytometry

The immunophenotyping test was carried out on whole blood. Leukocytes and lymphocytes subset were performed by multiparameter flow cytometry method with panel of monoclonal antibodies using FACS Canto II BD flow cytometry (Becton Dickinson, Franklin Lakes, NJ, USA). For surface markers detection on leukocytes and lymphocytes T, B, NK, Treg cells and activated Treg subset cells were stained with fluorescently labelled antibodies: CD4-FITC (catalog number: 345768, clone number: SK3), CD56-PE (catalog number: 345810, clone number: MY31), CD3-PerCP-Cy5.5 (catalog number: 332771, clone number: SK7), CD19-PE-Cy7 (catalog number: 341113, clone number: SJ25C1), CD8-APC (catalog number: 345775, clone number: SK1), CD16-APC-H7 (catalog number: 560195, clone number: 3G8), HLA-DR-V450 (catalog number: 655874, clone number: L243), CD25-APC (catalog number: 340907, clone number: 2A3), CD127-FITC (catalog number: 560549, clone number: HIL-7R-M21), CD45RO-PE-Cy7 (catalog number: 337168, clone number: UCHL1), CD95-PE (catalog number: 340480, clone number: DX2), CD4-PerCP-Cy5.5 (catalog number:332772, clone number:SK3), CD3-APC-H7 (catalog number:641415, clone number:SK7) and CD45-V500 (catalog number: 655873, clone number: 2D1), (BD Bioscience). Two test tubes were used for the different multicolor panel:

Tube 1: CD4 FITC, CD56PE, CD3-PerCP-Cy5.5, CD19-PE-Cy7, CD8-APC, CD16-APC-H7, HLA-DR-V450, CD45-V500

Tube 2: CD127-FITC, CD95-PE, CD4-PerCP-Cy5.5, CD45RO-PE-Cy7, CD25-APC, CD3-APC-H7, CD45-V500

Samples were incubated for 20 min in room temperature. After two washing, cells were analyzed within 2 h. For each sample, a minimum of 20,000 events were collected.

**Figure A2:**
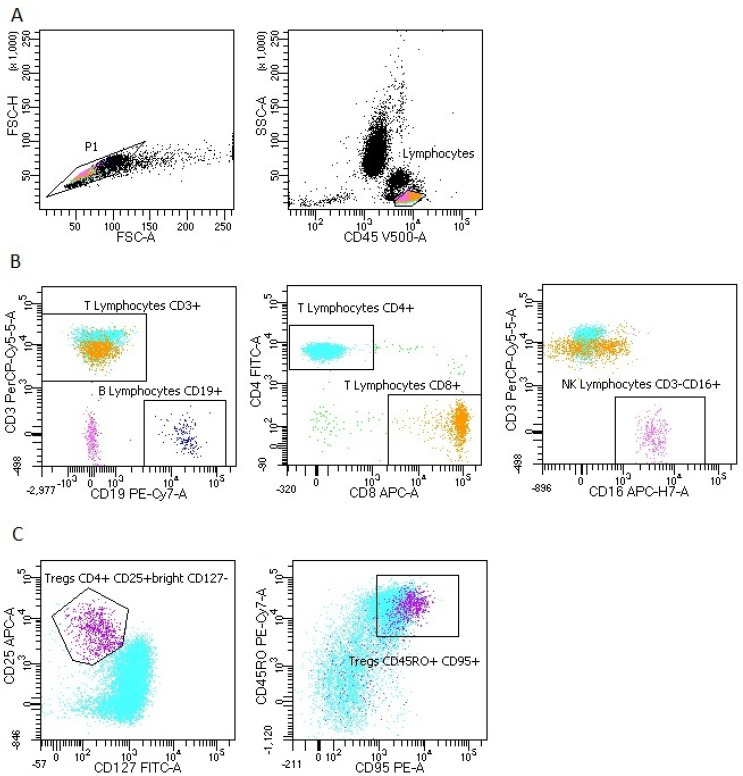
Representative FACS analysis of PB cells with antibodies specific for lymphocytes subpopulations and T regulatory (Treg) cells. (**A**) Lymphocytes gating strategy: FSC-A vs. FSC-H plot: Gating the cells that have an equal area and height, thus removing clumps (greater FSC-A relative to FSC-H) and debris (very low FSC), CD45 vs. SSC-A plot: Selection of lymphocytes based on their SSC/CD45+ properties. (**B**) Lymphocyte subpopulation gating strategy: CD3 vs. CD19 plot: Selection of lymphocytes T (blue and yellow) based on their CD3+ properties and lymphocytes B (navy blue) based on their CD19+ properties. CD4 vs. CD8 plot: Selection of T lymphocytes subsets CD4+ (blue) and CD8+ (yellow) based on their CD4/CD8 properties. CD3 vs. CD16 plot: Selection of NK cells (pink) based on their CD16+ properties and CD3 negative. (**C**) Treg cells and Tregs subsets gating strategy: CD25 vs. CD127 plot: Selection of Treg cells (purple) based on their CD25+high and CD127 negative properties. CD45RO vs. CD95 plot: Treg cells subset with CD45RO and CD95 expression.

The representative gating strategy of PB cells with antibodies specific for lymphocytes subpopulations and Treg cells was presented in Figure A2. 

Data were analyzed with DIVA Analysis software 8.0.1 (BD) and Infinicyt 1.8 Flow Cytometry (Cytognos, Salamanca, Spain).

### 2.6. Statistical Analysis

All statistical analyses were performed using the Statistica 13.0 software (TIBCO Software, Palo Alto, CA, USA). The results were expressed as means and SDs and medians with interquartile range (Q1–Q3). For group comparison the Mann–Whitney U test was used. For graphic processing was used Prism GraphPad (Version 7, GraphPad Software, La Jolla, CA, USA). Spearman rank test was performed to test correlations between variables. Statistical significance was determined as *p* < 0.05.

## 3. Results

**Figure 1:**
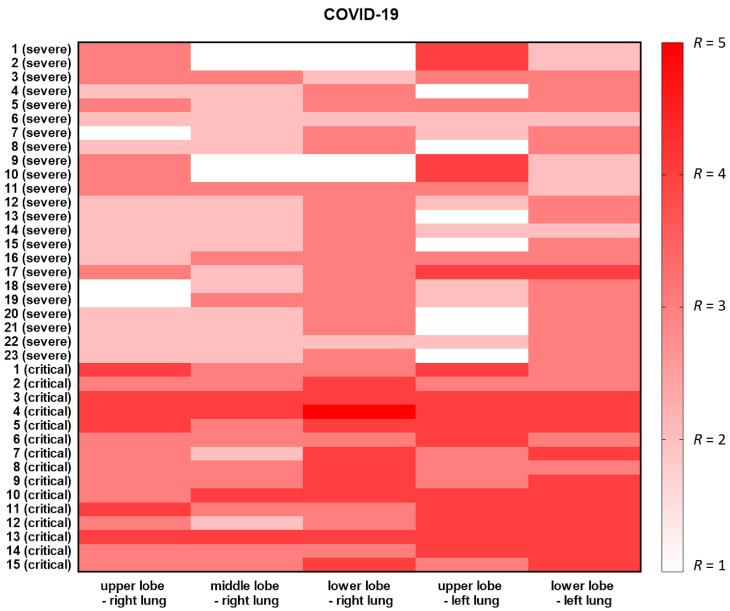
Heatmap with CT severity score in each patient. Lung lesion advancement grading was shown based on visual assessment of the degree of pulmonary tissue involvement by lesions in individual lobes (three lobes of the right lung and two lobes of the left lung). Each lobe was awarded a CT score from 0 (white) to 5 (red), depending on the percentage of the involved lobe.

Patients in our study were divided into two groups: severe COVID-19 group (*n* = 23 with CT severity score 11–15 and critical COVID-19 group (*n* = 15 with CT severity score 16–25) (Table 1). Heatmap with CT severity score in individual lobes (three lobes of the right lung and two lobes of the left lung) for each patient was presented in Figure 1.

The clinical characteristics of the investigated groups with severe COVID-19 and critical COVID-19 were summarized in Table 1. There were no significant differences between groups in age and the incidence of clinical signs of infection such as cough and fever. Dyspnea and respiratory failure occurred more frequently in critical patients. The oxygen saturation level SpO_2_ was significantly lower in critical group. Coexisting disorders such as diabetes, hypertension, obesity, coronary heart disease and neoplastic diseases occurred in both groups in individual cases. 

### 3.1. Basic Leukocytes Profile

The study groups were compared using the assessment of leukocyte subpopulations by flow cytometry (Table 2). We observed lower proportion of lymphocytes in critical COVID-19 patients than severe COVID-19 group (respectively, 10.3 vs. 23.1, *p* < 0.05). The proportion of T lymphocytes was also lower in critical COVID-19 than severe COVID-19 patients (respectively, 6.9 vs. 18.0, *p* < 0.05). The median proportion of CD4+ cells was lower in critical COVID-19 than severe COVID-19 patients (respectively, 3.8 vs. 11.3, *p* < 0.05).

The median proportion of Treg cells was lower in critical COVID-19 than severe COVID-19 patients (respectively, 0.214 vs. 0.557, *p* < 0.05). However, the proportion of Treg CD45RO+ CD95+ lymphocytes among Treg cells was higher in the critical COVID-19 patient than in the severe COVID-19 patient (81.3 vs. 76.5, *p* > 0.05)

When we analyzed the median proportion of B lymphocytes, we observed lower value in critical COVID-19 than severe COVID-19 patients (1.3 vs. 2.3, *p* < 0.05). The median proportion of NK cells was also lower in critical COVID-19 than severe COVID-19 patients (1.1 vs. 3.5, *p* < 0.05). 

We observed higher median proportion of neutrophils in critical COVID-19 than severe COVID-19 patients (85.3 vs. 67.3, *p* < 0.05). The neutrophil-to-lymphocyte ratio (NLR) was higher in critical COVID-19 than severe COVID-19 patients (8.4 vs. 2.9, *p* < 0.05) 

**Figure 2:**
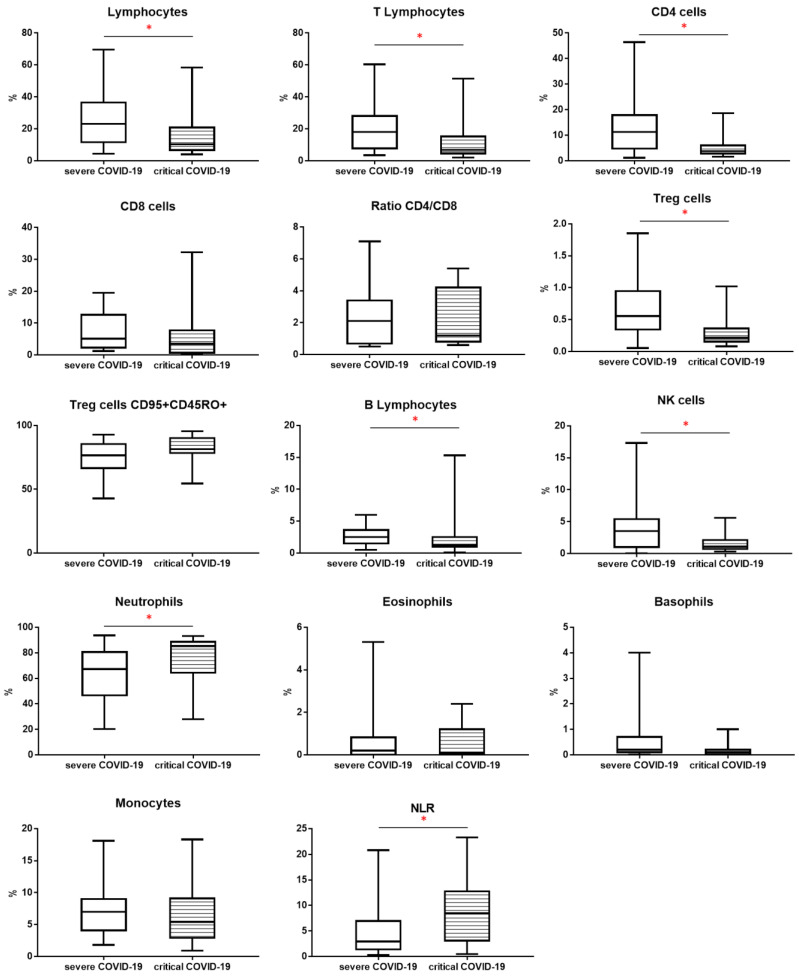
The differences of percentage of leukocytes, lymphocytes subpopulation and NLR between patients: with severe COVID-19 and critical COVID-19. Graphs show the median values (Min-Max) * *p* < 0.05. Lymphocytes, T, B, CD4, CD8, NK cells, T regulatory (Treg) cells, neutrophils, eosinophils, basophils and monocytes are presented as % of leukocytes. Treg cells CD95+ CD45RO+ is presented as % of Treg cells.

The above results and differences are presented in Table 2 and Figure 2. 

### 3.2. Cytokines Profile

The study groups were compared using the assessment of selected cytokines by Luminex technique which based on sandwich immunoassay that combines the enzyme-linked immunosorbent assay (ELISA) with flow cytometry. 

**Figure 3:**
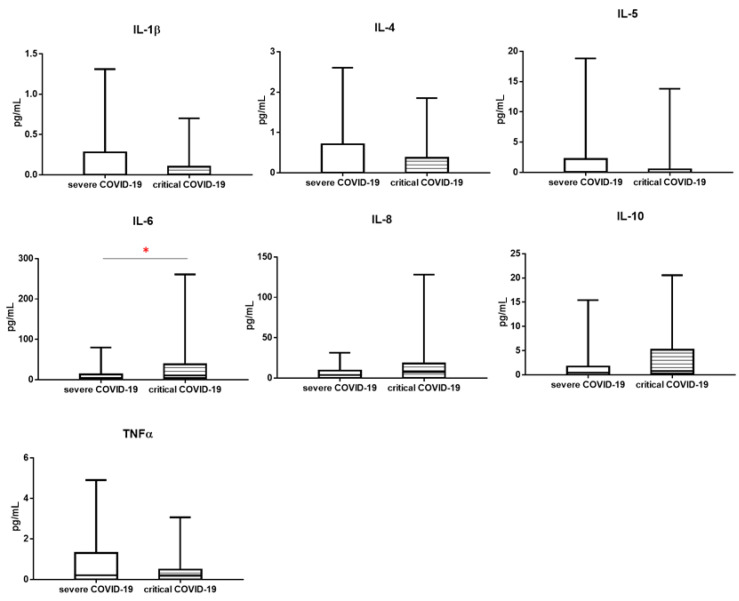
The differences of cytokines concentrations between patients: with severe COVID-19 and critical COVID-19. Graphs show the median values (Min–Max), * *p* < 0.05.

When we analyzed the median proportion of selected cytokines levels (pg/mL) such as: IL-1β, IL-4, IL-5, IL-6, IL-8, IL-10 and TNFα, we observed significant difference between severe COVID-19 and critical COVID-19 patient only for IL-6 level. The median proportion of IL-6 level was higher in critical COVID-19 than sever COVID-19 patients (respectively 10.5 vs. 4.0, *p* < 0.05), similar tendency was for IL-8 level, but not significant (7.6 vs. 3.9, *p* > 0.05). IL-10 also showed the same trend (0.831 vs. 0.460, *p* >0.05) (Table 3, Figure 3). 

### 3.3. Correlation between CT Severity Score and Study Parameters 

We used CT severity score to assess the progression of diseases in COVID-19 patients. Correlation analysis between CT severity score and laboratory data such as: study leukocytes subpopulation and cytokines was presented in Table 4. CT severity score was significantly negative correlated with proportion of lymphocyte, T lymphocytes, CD4+ cells, Treg cells and NK cells. We observed significant positive correlation between CT severity score and neutrophils, as for NLR parameter. IL-6 concentration was significantly positive correlated with CT severity score. Correlations between study parameters and CT severity score are presented in Table 4. 

### 3.4. Correlation between Cytokines Concentration and Leukocyte Subpopulations Depending on Severity of COVID-19

We also analyzed the correlation between the analyzed parameters, i.e., leukocyte subpopulations and cytokines concentration in two groups depending on the stage of the disease.

In the severe COVID-19 group we observed high positive correlation between the proportion of IL-10 level and neutrophils (*r* = 0.455, *p* = 0.0293), high negative correlation between proportion of IL-10 level and eosinophils (*r* = −0.515, *p* = 0.0118) and between proportion of IL-6 level and eosinophils (*r* = −0.475, *p* = 0.0219). Moreover the significantly negative correlations between the proportion of IL-6 level and Treg cells (*r* = −0.483, *p* = 0.0197) and between the proportion of IL-10 level and Treg cells (*r* = −0.463, *p* = 0.0262) were observed.

In the critical COVID-19 group we observed more significant correlation between leukocytes and study cytokines. Moreover in compared to the severe COVID-19 group we observed significant correlations between lymphocytes subpopulation and selected cytokines. There was significant positive correlation between proportion of lymphocytes and IL-1β (*r* = 0.591, *p* = 0.0204) and between proportion of lymphocytes and TNF-α (*r* = 0.615, *p* = 0.0148). We observed significant positive correlations between proportion of T lymphocytes and TNF-α level (*r* = 0.539, *p* =0.0382). CD8+ cells significantly positive correlated with proportion of IL-1β level (*r* = 0.550, *p* = 0.0335) and with TNF-α level (*r* = 0.604, *p* = 0.0172). There was positive correlation between proportion of lymphocytes B and IL-1β (*r* = 0.625, *p* = 0.0127). The significantly negative correlation between the proportion of IL-10 level and Treg cells was observed (*r* = −0552, *p* = 0.0328). NLR was significantly negatively correlated with proportion of IL-1β level (*r* = −0.591, *p* = 0.0203) and with TNF-α level (*r* = −0.615, *p* = 0.0148).

**Figure 4:**
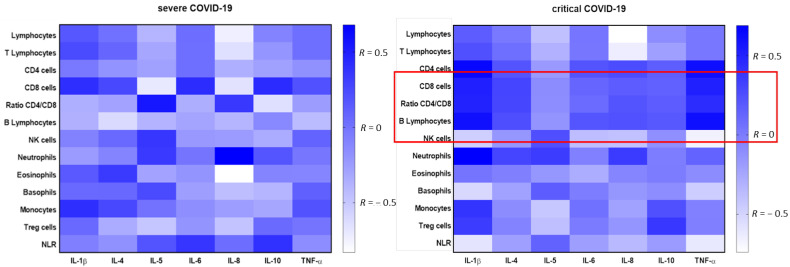
A heatmap of Spearman correlation coefficients for the cytokine levels with leukocytes using multiparameter flow cytometry in severe COVID-19 and critical COVID-19 group. Correlations with an absolute value more than 0.5 are associated with *p* < 0.05, blue—positive correlations, white—negative correlations. The red line highlights significant positive, strong correlations between the studied parameters in the critical group.

All correlations between study leukocytes subpopulation and cytokines in the 2 groups were presented on heat maps (Figure 4). The red line highlighted significant positive, strong correlations between the studied parameters in the critical group and indicated the lack of the same correlations in the severe group.

## 4. Discussion

We examined changes in peripheral blood leukocyte subsets and parallel alternation in cytokine levels in patients with different disease severity assessed on the basis of the CT severity score to explain the pathogenesis of SARS-CoV-2. We found that patients with critical COVID-19 had decreased lymphocyte values including: T lymphocytes, CD4+ cells, B lymphocytes and NK cells. As reported in recently published studies lymphopenia has been observed in most critical COVID-19 cases on admission and may be a potential prognostic factor [13,14,15,16]. In our previous study we found a lower proportion of T lymphocytes (CD4+ and CD8+ subsets), B lymphocytes, eosinophils and basophils in COVID-19 with interstitial lesions on chest X-ray than in healthy controls but we did not observe the differences in absolute number of analyzed leukocytes subpopulations between patients with and without lung lesions on chest X-ray [17]. This may confirm that the decrease in the absolute number of leukocytes may be related to the severity of the disease and manifest itself more in severe cases. The mechanisms of lymphopenia can include immune dysregulation due to cytokine accumulation which affects lymphocyte apoptosis, migration of immune cells into the lungs, and impairment of lymphoid organs [18,19]. Research by Vedder et al. may suggest migration of lymphocytes to the site of infection and explain peripheral blood lymphopenia. They evaluated cellular profile in bronchoalveolar lavage (BAL) in COVID-19 patients and observed increased CD8+ T-cell values in direct comparison to other Corona virus types [20]. It is unclear whether SARS-COV-2 can directly infect lymphocytes, which requires more detailed studies. The mechanism of lowering the percentage of peripheral Treg cells is also unknown. In our study, e observed a decrease in Treg cells proportion depending on the severity of the disease. The recent research suggests that the level of peripheral Treg cells is prominently reduced in severe COVID-19 patients compared with mild patients [21,22,23]. Wang, W. et al. [21] have shown that Treg cells increased during the progression from mild to severe condition but then declined during the progression to critical condition. Similarly Wang, F. et al. [23] have presented that the percentage of natural Treg cells was decreased in extremely severe patients. Additionally, we noticed that the phenotype of activated Treg cells CD45RO+ shifted towards enhanced apoptotic susceptibility by the high level of expression of pro-apoptotic molecule CD95 (Fas/APO-1). Increased apoptosis likely contributes to impaired survival of regulatory T cells and insufficient immunosuppressive function of these cells [24]. We hypothesize that apoptotic Treg cells may play an important role in the advanced stages of COVID-19. 

Similar to other studies increased percentages of neutrophils and NLR parameter have been noted in our study [25,26,27]. The NLR in critical patients was higher than in severe, but there is no systematic review and meta-analysis to assess predictive NLR values for disease severity. It is therefore unclear about the NLR thresholds that should be used to classify disease severity and predict prognosis.

It is known that patients with COVID-19 are unregulated immunologically with fast response to infection and high cytokine release. Researchers have shown that the severity of COVID-19 is associated with increased levels of inflammatory mediators, including cytokines and chemokines, such as interleukin IL-2, IL-7, IL-10, TNF, granulocyte colony stimulating factor (G-CSF), monocyte chemotactic protein-1 (MCP1), macrophage inflammatory protein 1 alpha (MIP1α) or CXC10 chemokine ligand (CXCL10) in the blood after SARS-CoV-2 infection [5,28,29]. In our study, when analyzing the differences in median levels of cytokines in patients with severe and critical COVID-19, we noticed significant changes only for IL-6 level. Others also found that serum IL-6 concentration is closely associated with the severity of COVID-19 disease [30,31,32]. They have shown that COVID-19 patients have higher serum level of cytokines: TNF-α, IFN-γ, IL-2, IL-4, IL-6 and IL-10 than control individuals. Within COVID-19 patients, only serum IL-6 and IL-10 levels are significantly higher in critical group than in moderate and severe group [33]. Jing Zhang et al. [32] also have observed that IL-6 concentrations were significantly increased in critical patients. They suggested that serum IL-6 level is a good marker of severity in patients with SARS-CoV-2 infection. 

Our study showed that using the assessment of the correlation of the CT severity score with various examined parameters showed the tendencies to decrease in leukocytes and increase in neutrophils percentages and NLR along with the stage of advancement. The CT severity score uses specific cut-off values to classify patients into specific advancement groups [10,11]. CT is an effective method of detecting abnormalities in the lungs, especially in the early stages of the disease and the high sensitivity of CT makes this method ideal for assessing the severity of the disease. However, the changes observed in CT examination in patients with SARS-CoV-2 infection are not specific enough to use tomography as a tool of definitive diagnosis [34,35].

Taking into account the above studies and our results, it seems important to search for parameters whose specific value would allow to determine the stage of the disease or to use combinations of several parameters to determine the specific profile of the disease severity. Although it has not been investigated whether cytokines and leukocyte subpopulations are directly involved in lung pathology during COVID-19, evaluation of changes in these parameters, including reductions in total lymphocyte counts, lymphocyte subsets and elevated neutrophils percentages, NLR levels, and IL-6 levels, was strongly correlated with the severity of the disease. In addition, we assessed the correlation of the studied parameters with each other and compared it between the groups with severe and critical COVID-19. We noticed correlation in the severe COVID-19 group between cytokines IL-6 and IL-10 levels and neutrophils, what was reported by other studies [31,36]. Dhar et al. [31] also indicates a possible dysregulation of the immune response against COVID-19, in which the two cytokines IL-6 and IL-10 play a main role. They suggest that measuring these cytokines could help identify patients more likely to progress to severe disease. In our study in the severe COVID-19 group we observed high negative correlation between proportion of IL-6 level and eosinophils. Eosinopenia had been found in numerous studies lately to have a strong correlation with COVID-19 mortality and along with lymphopenia may be a useful pointer for diagnosing COVID-19 in those patients [37,38,39].

In contrast to the severe COVID-19 group, the critical COVID-19 group was notable for the existence of more and different correlations between proinflammatory cytokines: TNF-α, IL-1β and cells, including lymphocyte subpopulations: T lymphocytes CD8+, B lymphocytes and Treg cells. We hypothesized that pro-inflammatory cytokines together with highly immunocompetent subpopulations of cells (T lymphocytes: CD4 and CD8 and B lymphocytes) play a significant role in the most advanced group, which was not observed in the sever group. In the critical group, there was a noticeable lack of significant correlations with neutrophils, eosinophils, which are the main components of the innate non-specific immune response. During innate immune response, pro-inflammatory cytokines, especially INF-γ, induced by neutrophils, monocytes, macrophages and dendritic cells are produced [40]. Severe COVID-19 cases had elevated the levels of various cytokines, including granulocyte colony stimulating factor, IL-10, TNF-α, MIP-1α and MCP-1 [41]. However, the exact role of innate immunity against COVID-19 is not fully understood [42]. In the adaptive immune response, CD4+ T cells perform a helper and effector function, CD8+ cells contribute to virus clearance by lysing infected cells meanwhile B cells produce virus-specific antibodies and neutralize the viruses [43]. It is known that the presence of T cells and antibodies is associated with successful resolution of average all cases of COVID-19 [44]. He, S. et al. [45] found a reduced number of CD4+ T, CD8+ T lymphocytes and increased levels of IL-6 and IL-10 in patients with advanced lung lesions had significantly fewer lymphocytes, and this decrease was negatively correlated with the area of lung lesions. It can be seen that SARS-CoV-2 mainly affected lymphocytes causing a deficiency of cellular immunity. Transient lymphopenia is a common feature of many respiratory viral infections, such as with influenza A H3N2 virus, respiratory syncytial virus or human rhinovirus, but in contrast to COVID-19 infection typically occurs for only 2–4 days around symptoms [46]. Peripheral lymphopenia in COVID-19 patients seems to be more selective for T lymphocytes and could reflect recruitment of lymphocytes to inflamed respiratory sites. However, infiltration of lymphocytes in the BALF was observed, but some researchers show that the number of these cells was not elevated [47,48]. 

It should be mentioned, that despite lymphopenia, immune cells respond to COVID-19 infection. In our previous studies, we showed an increase in the number of activated lymphocytes - RE-LYMP parameter and plasmablasts [6] as well as CD4+ central memory and CD8+ effector cells in patients with COVID-19 without interstitial lesions on chest X-ray and with interstitial lesions on chest X-ray compared to the control group [17]. Other researchers also point to the emerging immune response in the form of activated, effector and memory T cells depending on the severity of the disease [49,50]. Furthermore, not only activation receptors appear on lymphocytes, but also exhausted markers and receptor inhibitors are revealed [51,52,53] It is not entirely clear whether the expression of these receptors reflects over-activation or exhaustion of lymphocytes and requires further research.

Those above findings indicate a dysregulation of both innate and adaptive immunity, and intensity of these immunological changes could be related to the severity of the disease. Thus, direct research of innate and adaptive immunity including cytokine-leukocytes profile and its relationship to disease severity in SARS-CoV-2 infected patients could be crucial to understanding pathogenesis of COVID-19. In our opinion detailed CT results of lung lesions along with the cellular and cytokine profile will give a better clinical picture of the patient and may contribute to selecting the optimal and individual therapy.

## 5. Conclusions

Our study showed that differences between patients in the advanced stages of COVID-19 can be seen using a combination of cytokine levels and leukocyte subpopulation assessments. In addition, we noticed that in patients with critical COVID-19 there were more interrelationships in the cytokine-lymphocytic profile. The results of our study may help to better understand the role of the research profile consisting of image of pulmonary lesions measured by CT severity score, cytokines and leukocyte subpopulations and indicate a potential therapeutic target in patients with advanced COVID-19.

## Figures and Tables

**Table 1:** Patients’ characteristics.

	Severe COVID-19 *n* = 23	Critical COVID-19 *n* = 15
Sex: f/m (n)	15/8	1/14
Age (mean ± SD years)	54.9 ± 14.4	59.1 ± 12.0
Clinical symptoms (n, %)		
- fever	20, 87.0%	15, 100%
- cough	10, 43.5%	11, 73.3%
- dyspnea	8, 34.8%	14, 93.3%
- respiratory failure	5, 21.7%	13, 86.7%
Saturation (mean ± SD %)	94.0 ± 4.2%	88.2 ± 6.7%
Diseases comorbidities (n, %)		
- diabetes	2, 8.7%	4, 26.7%
- hypertension	3, 13.0%	7, 46.7%
- obesity	1, 4.3%	4, 26.7%
- coronary heart disease	3, 13.0%	3, 20.0%
- neoplastic diseases	2, 8.7%	3, 20.0%

Abbreviation: f, female; m, men; SD, standard deviation.

**Table 2:** Differences in the median of white blood cell (WBC) count, leukocytes and main lymphocytes subpopulation in patients with severe COVID-19 and critical COVID-19. Data expressed as median (Q1–Q3). * *p* < 0.05.

	COVID-19 Severe *n* = 23 Median (Q1–Q3)	COVID-19 Critical *n* = 15 Median (Q1–Q3)	Mann-Whitney U Test
WBC [k/µL]	5280 (4580–8620)	9220 (4370–13010)	* 0.0382
[% of leukocytes]			
Lymphocytes	23.1 (11.6–36.4)	10.3 (6.7–20.8)	* 0.0219
T Lymphocytes	18.0 (7.8–28.0)	6.9 (4.6–15.2)	* 0.0514
CD4 cells	11.3 (4.8–17.9)	3.8 (2.8–6.0)	* 0.0.018
CD8 cells	5.1 (2.3–12.6)	3.5 (0.7–7.7)	0.2238
Ratio CD4/CD8	2.1 (0.7–3.4)	1.2 (0.8–4.2)	0.8828
Treg cells	0.557 (0.346–0.947)	0.214 (0.155–0.360)	* 0.0061
Treg cells CD45RO+ CD95+ [% among Treg cells]	76.5 (67.1–85.2)	81.3 (79.1–89.0)	0.0959
B Lymphocytes	2.5 (1.5–3.6)	1.3 (1.0–2.5)	* 0.0412
NK cells	3.5 (1.0–5.4)	1.1 (0.7–2.1)	* 0.0238
Neutrophils	67.3 (46.6–80.6)	85.3 (64.4–88.6)	* 0.0444
Eosinophils	0.2 (0.0–0.9)	0.1 (0.0–1.2)	0.9295
Basophils	0.2 (0.1–0.7)	0.1 (0.0–0.2)	0.1143
Monocytes	7.0 (4.1–9.0)	5.4 (2.9–9.1)	0.1340
NLR	2.9 (1.4–6.9)	8.4 (3.1–12.7)	* 0.0258

Abbreviation: CD, cluster of differentiation; NK, natural killer cells; NLR, neutrophil-to-lymphocyte ratio; Treg cells, T regulatory cells, WBC, white blood cells count.

**Table 3:** Differences in the median of cytokine levels in patients with severe and critical COVID-19. Data expressed as median (Q1–Q3). * *p* < 0.05.

Cytokines [pg/mL]	COVID-19 Severe *n* = 23 Median (Q1–Q3)	COVID-19 Critical *n* = 15 Median (Q1–Q3)	Mann-Whitney U Test
IL-1β	0.000 (0.000–0.283)	0.000 (0.000–0.100)	0.7013
IL-4	0.000 (0.000–0.710)	0.000 (0.000–0.370)	0.2861
IL-5	0.128 (0.000–2.200)	0.000 (0.000–0.500)	0.2729
IL-6	4.036 (0.474–13.000)	10.500 (4.000–38.380)	* 0.0382
IL-8	3.900 (0.000–9.4447)	7.600 (0.000–18.010)	0.3140
IL-10	0.460 (0.000–1.730)	0.831 (0.300–5.200)	0.1623
TNF-α	0.209 (0.000–1.300)	0.212 (0.000–0.490)	0.5548

Abbreviation: IL, interleukin; TNF-α, tumor necrosis factor α.

**Table 4:** Spearman rank correlation coefficients between CT severity score and laboratory data: leukocytes subpopulation and cytokines. * Means *p* < 0.05.

	CT Severity Score (*n* = 38 )	
	*r*	*p*-Values
Lymphocytes [%]	−0.402	0.0123 *
T Lymphocytes [%]	−0.344	0.0339 *
CD4 cells [%]	−0.387	0.0162 *
CD8 cells [%]	−0.311	0.0572
Ratio CD4/CD8 [%]	0.061	0.7156
Treg cells [%]	−0.392	0.0148 *
Treg cells CD45RO+ CD95+ [%]	0.219	0.2046
B Lymphocytes [%]	−0.284	0.0829
NK cells [%]	−0.374	0.0205 *
Neutrophils [%]	0.352	0.0301 *
Eosinophils [%]	−0.030	0.8540
Basophils [%]	−0.232	0.1598
Monocytes [%]	−0.049	0.7689
NLR	0.390	0.0152 *
IL-1β [pg/mL]	0.003	0.9847
IL-4 [pg/mL]	−0.223	0.1775
IL-5 [pg/mL]	−0.026	0.8762
IL-6 [pg/mL]	0.351	0.0304 *
IL-8 [pg/mL]	0.124	0.4579
IL-10 [pg/mL]	0.105	0.5294
TNF-α [pg/mL]	−0.150	0.3680

Abbreviation: CT, computed tomography; IL, interleukin; NLR, neutrophil-to-lymphocyte ratio; TNF-α, tumor necrosis factor α, Treg cells, T regulatory cells.
